# Shared responsibility for managing fatigue: Hearing the pilots

**DOI:** 10.1371/journal.pone.0195530

**Published:** 2018-05-21

**Authors:** Jennifer L. Zaslona, Karyn M. O’Keeffe, T. Leigh Signal, Philippa H. Gander

**Affiliations:** Sleep/Wake Research Centre, Massey University, Wellington, New Zealand; Charite Medical University Berlin, GERMANY

## Abstract

In commercial aviation, fatigue is defined as a physiological state of reduced mental or physical performance capability resulting from sleep loss, extended wakefulness, circadian phase, and/or workload. The International Civil Aviation Organisation mandates that responsibility for fatigue risk management is shared between airline management, pilots, and support staff. However, to date, the majority of research relating to fatigue mitigations in long range operations has focused on the mitigations required or recommended by regulators and operators. Little research attention has been paid to the views or operational experience of the pilots who use these (or other) mitigations. This study focused on pilots’ views and experiences of in-flight sleep as the primary fatigue mitigation on long range flights. It also sought information about other fatigue mitigation strategies they use. Thematic analysis was used to explore written comments from diary and survey data collected during long range and ultra-long range trips (N = 291 pilots on three different aircraft types, 17 different out-and-back trips, and four airlines based on three continents). The findings indicate that the recommended fatigue mitigation strategies on long-haul flights (particularly in-flight sleep) are effective and well-utilised, consistent with quantitative findings from the same trips. Importantly however, the analyses also highlight areas that require further investigation, including flight preparation strategies in relation to the uncertainty of in-flight break allocation. There were two strategies for sleep prior to a flight: maximising sleep if pilots were expecting later breaks in the flight; or minimising sleep if they were expecting breaks earlier or at unfavourable times in the circadian cycle. They also provide a broader view of the factors that affect the amount and quality of pilots’ in-flight sleep, about which evidence has previously been largely anecdotal. The study underscores the value of including the views and experience of pilots in fatigue risk management.

## Introduction

Fatigue is inherent to long haul flight operations, with operators and regulators alike increasingly turning to fatigue risk management systems (FRMSs) in order to mitigate the risk associated with fatigue. Guidance material from the International Civil Aviation Organisation (ICAO) defines these fatigue risk management systems as ‘*a data-driven means of continuously monitoring and managing fatigue-related safety risks*, *based upon scientific principles*, *knowledge and* *operational experience* *that aims to ensure relevant personnel are performing at adequate levels of alertness’* [1]. Throughout the ICAO guidance material the importance of ‘*shared responsibility*’ between management, crew members and other support staff is emphasized. This shared responsibility is recognized as an important concept in the guidance material because fatigue is a whole-of-life issue that is affected by all waking activities and not only by work demands.

The primary fatigue mitigation strategy recommended for long haul operations in this guidance material is the use of in-flight sleep [2]. Most of the research conducted to date with pilots has focused on in-flight sleep duration and timing and on monitoring pilot fatigue and performance on specific flight routes. As a result, although in-flight sleep is known to be of poorer quality than sleep obtained on the ground [3, 4], the use of in-flight sleep to mitigate fatigue risk has become well-accepted as an effective mitigation strategy in this environment [5–10]. This is valuable research, however it primarily centers around mitigations determined by regulators and operators with little emphasis on the operational experience of the pilots themselves.

Very few studies have investigated pilots’ views on the use of in-flight sleep as a fatigue mitigation or the factors that affect it. The few studies that have were conducted prior to the introduction of newer rest facilities or aircraft and used closed answer questions to investigate the quality and duration of in-flight sleep [11, 12], offering the studied pilots limited opportunities to comment on other factors affecting their in-flight sleep and not addressing factors contributing to fatigue. A more recent study investigated the use of informal fatigue management strategies in a group of defense aviation personnel, but the study was not specific to pilots and a number of the strategies used by participants (such as task slowing, task rotation and load shedding/delegation) are not applicable on the flight deck [13].

Pilots’ perspectives on the recommended fatigue mitigations are important, particularly in light of the emphasis on shared responsibility in FRMSs. Most individuals familiar with the aviation environment are aware that in-flight sleep can be disrupted by different factors and that pilots may use additional fatigue mitigation strategies, but evidence in this area remains mainly anecdotal. The present analyses aimed to address this gap in the literature by analyzing pilots’ comments about in-flight sleep and fatigue mitigation strategies across a range of long range and ultra-long range flights.

## Methods

This paper combines the findings of two separate qualitative analyses that made use of de-identified questionnaire and sleep diary data that had been collected as part of five studies monitoring pilots’ sleep and performance [6–9, 14]. All five studies received ethical approval (Massey University Human Ethics Committee Southern A, application 08/68; Massey University Human Ethics Committee, application 11/74; Massey University Human Ethics Committee, WGTN Protocol 04/03; Washington State University Institutional Review Board, IRB # 10951), participants provided written informed consent and the de-identified data were approved for use in subsequent analyses. These studies included a total of 17 long-range and ultra-long range routes operated by four different airlines using three different aircraft types.

In all five studies, pilots were asked to complete a retrospective questionnaire prior to the start of their study period and then completed a prospective sleep diary during their study period. Data from the final item of the questionnaire (an open-ended comments section) forms the basis of the first analysis (pilots’ perspectives on in-flight sleep), while pilots’ responses to the sleep diary question ‘How do you typically manage fatigue on this flight?’ forms the basis of the second analysis (pilots’ fatigue mitigation strategies). Pilots were asked to answer the question relating to how they managed fatigue for each studied flight segment (2–4 depending on the study), however this question was not included in the diaries of the study from 2004 [8], resulting in only 4 of the 5 studies being included in this second set of analyses.

All of the pilots’ written responses were analyzed using accepted methods of thematic analysis [15, 16], which include a 6-phase iterative process of coding, categorization and review. For the analysis of pilots’ perspectives on in-flight sleep (first analysis) this was completed in three stages (stages 1,2, & 4 below) by three researchers (the third acting in an advisory capacity). For the analysis of pilots’ fatigue mitigation strategies (second analysis) this was completed in four stages by two researchers as outlined below:

1. Familiarization with the data: the researchers independently read through the comments and each identified meaningful units of text (codes) based on recurring or interesting content (e.g.; ‘keep hydrated’, ‘flight attendant noise’).
2. Double coding: to ensure reasonable agreement of the coding, codes for each response were reviewed as a group. Where codes differed between researchers, these were discussed and edited to reflect what was agreed.
3. Finalizing coding definitions: coding definitions from each researcher were reviewed as a group and agreed final definitions were established for each individual code.
4. Identifying themes: the defined codes were grouped into themes (e.g.; ‘pre-flight habits’, ‘sleep environment), reviewed, and then regrouped into broader themes.

In both cases the analysis was primarily an experiential and inductive form of thematic analysis conducted within a pragmatic framework. As such, the themes explored in these analyses focused on participants’ experiences or perspectives and were generated based on what patterns were recognized in the data, instead of being constructed from an existing theory [17]. The coding process used in these analyses was descriptive and semantic; the researchers did not attempt to go beyond the participants’ meanings during the coding phase of analysis [15].

## Results

### Pilots’ perspectives on in-flight sleep

In total, data from 291 pilots were eligible for inclusion in this analysis. Of these, 138 (47.4%) provided written comments and response rates differed by study (range 26.8% to 62.9%). Fifteen crew members were excluded from the analyses as their comments had been abridged during data entry and the original questionnaires were not available for review at the time of analysis, as they were collected by a different research team and are archived at a different institution. Thus, this analysis includes comments from 123 pilots (refer to S1 Table for further detail of frequency of responses). Comments varied in structure from sentence fragments to long narratives.

Seven main categories of factors relating to in-flight sleep were identified and are presented in Table 1 below. The most common themes in the comments related to categories 1, 2 and 3 and pilots often linked these themes to those from category 4. The themes from these categories are the ones focused on here.

**Table 1:** Categorisation and frequency of occurrence pilots’ comments relating to in-flight sleep.

Category number	Perspectives on in-flight sleep	Number of comments relating to theme	%
**1**	Type, design & location of crew rest facility (work factor)	66	53.7
**2**	Personal factors affecting in-flight sleep	24	19.5
**3**	Timing & choice of rest breaks (work factor)	17	13.8
**4**	Impact on in-flight waking function	27	22.0
**5**	Concerns about flight safety	3	2.4
**6**	Use of flight deck napping	3	2.4
**7**	Impact of commuting	7	5.7

In their comments about in-flight sleep, pilots made it clear that they make good use of their in-flight rest opportunities with comments such as *“I always attempt to sleep”* but they also highlighted a number of factors which impacted on their in-flight sleep. In particular, they reported that the type, design and location of the crew rest facility can adversely affect in-flight sleep duration and quality, with some pilots adding that this led to decreased alertness, increased fatigue and could potentially decrease flight safety. Pilots made it clear that they preferred to use a crew bunk over a crew rest seat commenting that *“the chair sleep is a joke*. *Not dark*, *quiet or comfortable”* and that *“*[the] *Bunk is far superior”*. They felt that sleep in the crew rest seat was much more disrupted, which negatively impacted on their fatigue and alertness levels when they returned to the flight deck:

“I flew the 767 in 2000–2001 and took crew rest in a business class seat in the cabin. I never felt rested. The bunk on the 777 makes a huge difference and I feel awake and alert during all my cockpit duty periods”

Crew rest seats are located in the passenger cabin, which makes them less private and noisier than the crew bunks due to passenger and cabin crew noise and movement, with pilots describing that *“People bump you in the seat*, *move the curtain*, [and] *it’s noisy on 1*^*st*^ *and 3*^*rd*^ *break”*. Pilots also indicated that the quality of their sleep in the bunk was far better than in the crew rest seat with one individual explaining:

“Having flown the 757/767 for the previous 4yrs prior to the 777 and having mainly coach seats for rest (with an occasional First class seat for rest) I can attest that the rest I receive in the bunk, even though it may not seem long, is vastly superior to the ‘park bench’ rest I received on the 767”

Although pilots clearly preferred the crew bunk, they also had numerous complaints about it. The bunk was depicted as *“a closet with bunk beds”* where some pilots reported being able to hear *“noise from* [the] *galley & cockpit and especially the call chime from PAX* [passengers] *to galley the same as crew call”* while the sleeping surface was described as being uncomfortable to the point that it is *“in some way*[s] *like sleeping on a well padded carpet over hard floor”*.

In response to the disturbances experienced, pilots indicated that they had developed personal strategies, such as specific rest routines or the use of earplugs (e.g.; *“I wear earplugs while in crew rest and that helps a lot”*), to make the most of their in-flight sleep opportunities. Other personal factors were also reported to affect in-flight sleep, with one pilot explaining that *“The bunk experience varies*. *I seem to be getting better at adapting to it”*.

Pilots indicated that the timing and allocation of rest breaks impacted on their in-flight sleep and alertness, with remarks such as *“the sleep quality in the bunk is much better when accomplished during my normal body clock sleep hours*. *This significantly improves alertness during duty times”*. In particular, pilots indicated that prior knowledge of the rest breaks they would be allocated was important and that uncertainty of break allocation was more disruptive to the in-flight sleep and wakefulness of more junior pilots, explaining that:

*“The most critical part of being able to sleep in the bunk for me is to know what breaks to plan on before I show up for work*. *Generally the* [relief pilot] *position picks breaks after the Captain and First Officer therefore*, *the* [relief pilot] *might not be set up correctly for rest (ie should be tired because will be going on break first or vice versa) and will have a very tough flight from a proper rest standpoint”*

### Pilots’ fatigue mitigation strategies

Data from 749 flight segments (operated by augmented flight crews monitored during 4 different studies) were eligible for inclusion in this analysis. Of these, responses to the question of interest were provided for 629 flight segments (84%) (for further information relating to the frequency of responses, please refer to S1 Table). The majority of responses were in the form of sentence fragments. Five main categories of fatigue management strategies were extracted and are outlined in Table 2 below.

**Table 2:** Categorisation and frequency of occurrence of fatigue management strategies identified by pilots.

Fatigue management strategy	Number of related codes	Occurrence of related codes	%
In-flight rest opportunities	39	323	30.3
Flight preparation	62	345	32.3
Improving sleep and alertness in flight	34	310	29.1
Aligning body clock	18	75	7.0
Tough it out / fatigue isn’t an issue	3	14	1.3
Total	156	1067	100

Most pilots indicated that they managed their fatigue on the studied flights by making use of “*crew rest*” and attempting to “*sleep on* [their] *rest break*”. However, there was considerable variability in the organisation of the in-flight rest breaks. This was often associated with specific break preferences (e.g.; “*I prefer first break when available*”), habits for break use (e.g.; “*On 1st break I do not sleep so I am tired for 2nd break*”) and patterns of break allocation (e.g.; “*SLLS* [short long long short] *breaks selected by flying crew*”). Comments also tended to indicate that the organisation of breaks was designed to favour the landing crew with arrangements such as “*short long short long to get landing crew in on long break*” and break allocations, such that relief crews “*generally get 2nd choice*”.

Flight preparation was another fatigue mitigation strategy reported by pilots with some aiming to get “*lots of sleep*” and attempting “*to minimize time awake before reporting to duty*”, while others tried to increase the likelihood of obtaining good quality sleep in flight by trying to “*not take a nap at home*” or to “*stay up and active all day*”. In most cases, those who attempted to maximize their sleep prior to the flight planned on flying first and resting later in the flight, explaining for instance that they “*try to start* [the] *trip well rested and take last break to be alert for both departure and arrival*”. In contrast, pilots who expected to be allocated the earlier rest periods tended to rely more heavily on in-flight sleep as a fatigue mitigation strategy and reported planning to “*report somewhat tired in order to sleep well on first break*”. This preparation strategy also applied to pre-trip napping, with pilots explaining “*pretrip nap if I am first crew flying*. *No pretrip* [nap] *if I am first crew in rest facility*”.

An important component of these flight preparation strategies was prior knowledge of rest break allocation as these opposing flight preparation techniques frequently resulted from pilots planning their rest around an anticipated or desired pattern of in-flight rest. Pilots explained that “*Fatigue mgmt* [management] *usually start*[s] *before flight based on whether I'm relief crew or primary crew*” and that they “*recommend crews know prior to report if they are A or B crews*, [it] *is vital to preparing for trip and being well rested for flight*”.

Pilots also reported using different strategies to maintain their alertness between rest breaks and minimise disruptions during their breaks. For instance, in order to reduce sleep disruption, pilots used techniques such as limiting their liquid intake and “*avoid*[ing] *caffeine*” so that they could “*sleep all the way thru each rest break*”. They also indicated using strategies such as the use of “*earplugs & eye mask*” and “*ensur*[ing] *that bunk area is cool and comfortable*” to help create an environment more conducive to good quality sleep. Techniques to improve alertness during duty periods such as the “*strategic use of caffeine*”, “*conversation*” and use of “*bright lights in cockpit*” were also frequently reported.

Strategies relating to the timing of rest breaks and sleep on layover also emerged. Some pilots indicated that they “*try to sleep on normal body clock time*” or tried to select their rest breaks “*so as to keep as normal a sleep cycle as possible inflight*”, while others preferred to try to “*adjust to local time sleep pattern*”. As in the analysis relating to pilots’ perspectives on in-flight sleep, some indicated that obtaining sleep on certain rest breaks was difficult “*due to body clock*”.

Finally, there were a few comments indicating that, at least for some individuals, managing fatigue on the studied flights was not an issue, with one pilot explaining “*This is actually a very easy flight with regards to fatigue*. *The scattered rest periods make it easy to manage fatigue*. *Just as you get tired your next rest period is there again*”.

## Discussion

The novelty in these findings lies not in the views expressed by the pilots but rather in the systematic documentation and analysis of these views across a range of flight routes, aircraft types and operators. To the best of our knowledge, pilots’ views on in-flight sleep and the fatigue mitigations they use on long-haul flights have, to date, never been investigated using open-ended questions across a sample of this size and any evidence has remained largely anecdotal. These analyses are important in the context of shared responsibility in fatigue risk management, since pilots’ operational experience contributes valuable information that quantitative studies of fatigue may overlook, particularly when research questions are very specific. The documentation of the factors pilots consider affect in-flight sleep, and of the fatigue mitigation strategies they use across such a range of aircraft types and flight routes, have revealed similarities in pilots’ perceptions and provides a clearer picture of how the current practices and recommendations can be improved, highlighting the areas in which further research (quantitative and qualitative) is required.

There are of course limitations to the present analyses. Aviation field studies are generally designed to answer safety-related questions and greater importance is placed on quantitative measures of pilot functioning than on pilots’ perceptions. This was the case in the five monitoring studies included here for which the qualitative data were collected as a supplementary source of information. The data did not address pilots’ views on collaboration and communication with superiors and airlines in relation to Fatigue Risk Management. This is an important issue to be addressed in future studies. Additionally, due to the design of the studies from which these data were drawn, the representativeness of the participating pilots in relation to the total pool of eligible pilots cannot be assessed. In the five original studies, all pilots who were flying the trips studied during the data collection periods were eligible to participate. The number of pilots recruited in each study was related to the power calculations for the particular study design.

### Pilots’ perspectives on in-flight sleep

In their comments about in-flight sleep, pilots made it clear that they make good use of their in-flight rest opportunities, a finding that was supported by quantitative analyses of the same data sets [6–9, 14]. However, they also highlighted a number of factors which impacted on their in-flight sleep and linked in-flight sleep to their fatigue, alertness and performance across the flight.

In particular, pilots indicated that the type, design and location of the crew rest facility could adversely affect in-flight sleep duration and quality and that they preferred to use a crew bunk where available. When using the crew rest seat, the lack of privacy and noise from passengers and cabin crew, as well as cabin lighting, the size of the seat and the reclined position in which pilots must sleep, can impact on their comfort as well as their ability to fall asleep and stay asleep, particularly in seats which recline less than 40 degrees from vertical [18]. A previous study comparing in-flight sleep taken in a crew rest seat to sleep taken in a bunk, found that a greater proportion of pilots were able to obtain sleep in the bunk and that the sleep obtained in the bunk was of longer duration than that obtained in the crew rest seat. This led to greater improvements in performance and alertness levels [19], a finding that is echoed in pilots’ complaints about the crew rest seats and enthusiasm about the bunk facilities.

Despite pilots’ overwhelming preference for the crew bunk, there were also numerous complaints about the bunk facilities, which highlighted possible areas for improvement. The most common complaints about the bunks related to noise (caused by both passengers and cabin crew), the general comfort of the rest facility (e.g., mattress, pillow, temperature), and turbulence. Uncomfortable or inadequate bedding, as well as the dry atmosphere of the bunk facility, are all reported in prior studies to contribute to disturbed sleep [11, 12]. Although sleep disturbances resulting from atmospheric turbulence are difficult to address, sleep in the bunk might be improved by addressing some of the other factors known to affect in-flight sleep. Examples include simple changes, such as providing pilots with softer mattresses and a choice of pillows, along with more substantial modifications of the crew rest facilities such as enhanced sound-proofing, or locating the facility further from galleys, which could all help make the bunk more conducive to good quality sleep. Noise disturbance is known to effect the structure of sleep and can result in increased fragmentation of sleep and spending more time in the lighter stages of sleep (reviewed in [20]).

Pilots also reported that break timing was a significant issue affecting sleep in flight. Prior sleep history, time awake and the circadian body clock, all affect sleep duration, quality and timing [21, 22], leading to ‘windows’ of sleep opportunity across the biological day. As a result, pilots may find it more difficult to obtain sufficient good quality sleep on breaks scheduled during their biological day (when there is a high propensity for wakefulness) or if the rest break occurs shortly after another sleep episode. Previous studies have found that sleep duration and quality are affected by both the timing of the flight and the timing of the scheduled in-flight rest periods and that bunk sleep has beneficial effects on complex task performance [23, 24], a finding which echoes pilots’ observations about the quality of their in-flight sleep and their subsequent alertness.

Related to the timing of rest breaks is the choice of rest breaks. Pilots indicated that flight preparation based on expected or chosen rest breaks affected their in-flight sleep and that prior knowledge of the rest breaks they would be allocated was important, an issue that they discussed further in their comments about fatigue mitigation strategies.

All of these comments are important in ensuring that the operational experience of pilots is taken into account, particularly as they highlight factors which affect their in-flight sleep (the primary recommended fatigue mitigation strategy on these flights) that have not previously been included in the quantitative monitoring studies of fatigue on the routes included in these analyses. This is reinforced by the findings of the second thematic analysis.

### Pilots’ fatigue mitigation strategies

Similarly to the first analysis, the majority of pilots indicated that they managed their fatigue in accordance with ICAO recommendations, by attempting to sleep during their scheduled rest breaks (evidence of which can be seen in the quantitative analyses of the same data sets [6–9, 14]).

Although the methods and patterns of in-flight rest allocation varied across the sample, rest break allocations tended to favour the landing crews. As in previous studies, pilots indicated that relief crews are often allocated the least favourable or desirable rest breaks [12]. The strategy of allocating rest breaks to benefit the alertness of the landing crew during the final phases of flight is now incorporated into Federal Aviation Administration regulations for augmented flights which require that “Two consecutive hours in the second half of the flight duty period are available for in-flight rest for the pilot flying the aircraft during landing” [25]. This new regulation precludes a number of rest break patterns observed in the analysed sample and effectively requires altering some established rest break allocation procedures. However, as this regulation was not in place at the time of data collection, it would not have affected the pilots in this sample who indicated that rest break allocation impacted on fatigue management because it influenced their flight preparation.

Indeed, flight preparation was commonly reported as a fatigue management strategy, with two opposing approaches emerging. While some pilots tried to maximise their sleep prior to the flight by sleeping in on the day of their trip and minimising their time awake prior to duty, others attempted to increase the likelihood of obtaining good quality sleep in flight by increasing their time awake prior to duty. These differences were often linked to anticipated patterns of in-flight rest and were particularly pronounced in relation to pre-flight naps. This strategy makes use of the pressure for sleep which builds across the waking hours [26]. Pilots who anticipated flying first aimed to reduce the pressure for sleep by sleeping before their duty period, while those who expected to sleep first allowed the sleep pressure to accrue in order to more easily obtain sleep in flight (particularly on rest breaks that occurred during their normal waking hours). Currently, scientific knowledge of the effects of pre-trip naps on in-flight sleep during different flight operations is insufficient to establish general guidelines about the implementation of these naps. While a pre-trip nap prior to an evening departure does not affect in-flight sleep duration or quality, either during the first rest period or across the flight as a whole [7], it is also clear that sleep during in-flight rest breaks is affected by both the timing of the flight and the timing of the rest breaks in relation to the flight [23, 24, 27].

In this respect, and in the context of shared responsibility for fatigue risk management, it is important for crews to know their in-flight rest break pattern well ahead of time, as it may influence the way in which they manage their sleep prior to the flight, and pilots indicated that uncertainty in the rest break allocations made it more difficult for them to prepare adequately. Although prior knowledge of rest break patterns is an important contributor to flight preparation, pilots are discouraged from relying too heavily on in-flight rest and encouraged to obtain as much sleep as possible prior to their trip, as there is always a possibility that (for operational or other reasons) no in-flight sleep is obtained. In such a scenario, pilots who restricted their sleep prior to the flight might have their fitness for duty compromised by fatigue.

Additional fatigue management techniques reported by pilots were strategies to improve their in-flight sleep and alertness. In-flight sleep is known to be of poorer quality and more disrupted than sleep on the ground [3, 4, 11, 12] and pilots indicated that they had developed a number of strategies to minimize these disruptions, such as limiting their liquid intake and making the bunk environment more conducive to good quality sleep by using earplugs and/or eye masks.

Responses indicated that pilots were aware of circadian fluctuations in sleep propensity and waking function and that this made it more difficult for them to obtain sleep on certain rest breaks [12]. Some pilots manipulated their sleep timing in flight (by choosing specific breaks) and on layover in an attempt to continue sleeping at their usual times, while others preferred to try to adapt to their layover time zone. This is consistent with findings from a previous study where a number of pilots shifted their sleep timing on layover despite being advised to remain on their home time zone [28].

Finally, a small group of pilots reported that “*Fatigue is never an issue*!” or that they just “*live with it*” but it was unclear from some of their responses what, if any, fatigue mitigation strategies they used. Encouragingly, the first attitude appears to suggest that the fatigue mitigation strategies currently in place on these operations are effective, which aligns with the findings of previous quantitative studies in which in-flight sleep had beneficial effects on pilot performance and alertness levels at the critical end of flight phases [5, 28, 29]. However, the contrary “*tough it out*” attitude raises the question of whether these fatigue mitigations are sufficient and highlights the importance of educating pilots on the negative effects of fatigue and on personal strategies that they can use in flight.

## Conclusions

In the context of shared responsibility for managing fatigue on these flights, it is important that the voices of pilots be heard as they provide different knowledge and experience which adds to the scientific principles and knowledge which shape fatigue risk management systems. It is therefore important in this context that pilots be willing and able to report fatigue hazards as outlined in ICAO guidance material [30]. This is encouraged by the use of non-punitive fatigue reporting systems, which distinguish between intentional violations (which are a disciplinary issue) and errors, which are a normal subset of human behaviour that becomes more likely with fatigue, and are a safety issue.

Overall, the findings indicate that the recommended fatigue mitigation strategies on long-haul flights (in particular the use of in-flight sleep), are effective and well-utilised, which aligns with the findings of related quantitative monitoring studies of sleep. Importantly though, these analyses also highlight areas of research (including flight preparation and break allocation) that require further investigation, as well as providing a broader understanding of the factors affecting in-flight sleep for which evidence has previously been primarily anecdotal.

## Supporting information

S1 Table Frequency of responses to the questions of interest by study. (DOCX)
