# Supplementary material for: Shared responsibility for managing fatigue: Hearing the pilots
Source: PLoS One. 2018 May 21;13(5):e0195530. doi: 10.1371/journal.pone.0195530 (PMC5962095; doi:10.1371/journal.pone.0195530)
Supplement: S1 Table — (DOCX) [file pone.0195530.s001.docx]

S1 Table. Frequency of responses to the questions of interest by study.

| Airline | Study description | Total number of participants | Number of valid questionnaires | Number who completed the comments section | Monitored flight segments per participant | Number of valid flight segments^b^ | Flight segments for which the fatigue management question was answered |
| --- | --- | --- | --- | --- | --- | --- | --- |
| Delta Air Lines | Monitoring study comparing pilots’ sleep and fatigue on long range (LR) vs. ultra-long range (ULR) flight operations | 70 | 70 | 44 (62.9%) | 4 | 279 | 232  (83.2%) |
| Delta Air Lines | Monitoring study comparing pilots’ sleep and fatigue between a full-size vs. tapered on-board bunk | 54 | 54 | 17 (31.5%) | 2 | 70 | 59 (84.3%) |
| Singapore Airlines | ULR flight validation (monitoring study) | 41 | 41 | 11 (26.8%) | 2 | N/A^c^ | N/A^c^ |
| South African Airways | ULR flight validation (monitoring study) | 52 | 52 | 22 (42.3%) | 2 | 104 | 82 (78.8%) |
| United Airlines | Monitoring study comparing pilots’ sleep and fatigue on LR vs. ULR flight operations | 74 | 59^a^ | 29 (39.2%)^a^ | 4 | 296 | 256 (86.5%) |
| Totals | | 291 | 276 | 123 | N/A | 749 | 629 (84.0%) |

^a^ The comments of 15 pilots were abridged during data entry and unavailable at the time of data analysis, these questionnaires were therefore excluded. The response rate for this study when including the abridged questionnaires is 59.5%.

^b^ Not all of the monitored flight segments met the criteria for inclusion in the original quantitative analyses (e.g., due to operational delays, data loss, flight diversions, …). In the qualitative analyses presented here, these invalid flight segments were also excluded.

^c^ The question relating to pilots’ fatigue mitigation strategies was not included in the Singapore Airlines study therefore this study was not included in the analysis of pilots’ fatigue mitigation strategies.
